# Incidence of Influenza Virus Infection among Wroclaw’s Healthcare Workers in Pre-COVID-19 2019–2020 Influenza Season Using Novel Flu SensDx Device

**DOI:** 10.3390/ijerph19063159

**Published:** 2022-03-08

**Authors:** Michał Jacek Jędrzejek, Agnieszka Mastalerz-Migas, Paulina Janicka

**Affiliations:** 1 Department of Family Medicine, Wroclaw Medical University, W. Syrokomli 1, 51-141 Wroclaw, Poland; agnieszka.mastalerz-migas@umed.wroc.pl; 2 Department of Pathology, Wroclaw University of Environmental and Life Sciences, Norwida 31, 50-375 Wroclaw, Poland; paulina.janicka@upwr.edu.pl

**Keywords:** influenza, vaccination, healthcare workers, point-of-care test, Flu SensDx

## Abstract

Background: Healthcare workers (HCWs) are more exposed to influenza infection, and the influenza vaccination is recommended each year, to reduce the risk of influenza infection and prevent influenza transmission. This study is a cross-sectional study and the objectives were to determine the rate of influenza virus infection among HCWs in the 2019–2020 influenza season. Methods: Between January and March 2020, a survey was carried out in 2 hospitals and 15 primary health-care settings (PHCS) in Wroclaw (Poland). The novel point-of-care testing Flu SensDx device was used, which detects the M1 protein of the influenza virus using electrochemical impedance spectroscopy from biological material (throat/nasal swabs). Results: A total of 150 samples were collected. The majority of participating HCWs by profession were 83 physicians (55.3%) and half (51.3%) of the participating HCWs worked in PHCS. Influenza vaccination coverage was 61.3% in 2019–2020 and 46.0% in the 2018–2019 season for all participants. Of the participating HCWs, 44.0% were positive tested by the Flu SensDx device. There were no statistically significant differences among the positive tested HCWs, their influenza immunization history, and the presence of symptoms of influenza-like illness (*p* > 0.05). Conclusion: Although the results of the present study suggest that influenza vaccination does not reduce the frequency of influenza virus detection by Flu SensDx testing in the HCWs participants, larger studies are needed to estimate the incidence of influenza virus infection among HCWs to understand the underlying mechanism and fine-tune policies aimed at reducing nosocomial infections.

## 1. Introduction

Influenza (flu) is an acute respiratory infection (ARI) of viral etiology, with a potentially severe and fatal course, especially for children, pregnant women, the elderly and people with chronic diseases [1]. The published data support the hypothesis that healthcare workers (HCWs) can act as a vector for the spread of influenza among hospitalized patients [2,3] and at the same time, medical personnel is more exposed to influenza infection (up to 2.5 times) compared to the population of healthy adults working in establishments other than healthcare facilities [4]. According to the empirical data, laboratory-confirmed frequency of influenza infection among HCWs could vary from 23.2% [5] to 29% [6]. Moreover, the results of various studies show directly that a large group of physicians (even more than 75% [7]) admits that they perform their professional duties while having symptoms of ARI (presenteeism) [8,9,10]. In this way, HCWs can introduce the influenza virus and perpetuate its transmission, putting patients at risk; thus, the phenomenon of healthcare-associated influenza (HAI) is becoming increasingly important in the literature. Although hospital influenza epidemics occur in almost all types of wards and have significant consequences for patients, the source of infection is often unknown [11]. It is postulated to implement systematic laboratory epidemiological surveillance as a key element in the practice of controlling influenza virus transmission in healthcare facilities.

In clinical practice, according to Polish recommendations, during an epidemic season of influenza, the classic symptoms are sufficient to diagnose influenza or influenza-like-illness (ILI), i.e., fever (>37.8 °C), myalgia, headache, fatigue, dry cough and sudden onset of symptoms [1]. Laboratory diagnostic is not a routine procedure and it may be performed in uncertain cases or off-season (in Poland, in the context of General Practice [GP] these procedures are not financed by national health services). Serological diagnostic is impractical; it is only used in scientific studies, and other methods, i.e., viral culture or the detection of the genetic material of the influenza virus by real-time reverse transcription polymerase chain reaction (RT-PCR), as a “gold standard” in influenza diagnosis, are expensive and require specialized laboratories and trained personnel [1]. Rapid influenza diagnostic tests (RIDT) may be an alternative in outpatient diagnostic procedures, but due to their low sensitivity and specificity, their value is limited and not widely used. There is a need for a novel point-of-care test (POCT) with high sensitivity and specificity.

From 2019, a new type of RIDT is available for wide use, i.e., the Flu SensDx device (SensDx S.A., Gdansk, Poland), which qualitatively detects the M1 protein of influenza virus (direct detection by bioreceptors), using electrochemical impedance spectroscopy (EIS), without differentiating between type A and B of the virus. The Flu SensDx device includes the following components: swab (Copan Flock), buffer (Flu Bufor SensDx), the Flu SensDx microsensor and platform (MOBI SensDx). All elements that make up the Flu series were registered on 20 December 2018 at The Office for Registration of Medicinal Products, Medical Devices and Biocidal Products (pol. Urząd Rejestracji Produktów Leczniczych, Wyrobów Medycznych i Preparatów Biobójczych). According to the producer’s information, validation tests were carried out and the specificity and sensitivity of the Flu SensDx test (in comparison to the RT-PCR test as a “gold standard”) were assessed at 96.97% and 91.67% respectively. It seems that this kind of RIDT may be helpful in clinical practice, but there is a need for a larger clinical validation of this device [1].

There are limited data on key aspects of influenza virus transmission in the community or healthcare settings and according to WHO experts and other authors, an extension of current knowledge is critical for developing evidence-based efficient strategies to control influenza virus transmission [3,12]. In the literature, the authors draw attention to the complexity of determinants of nosocomial infections and point to the need for research to estimate the burden of ARI, especially influenza, in the HCWs group and determine their role in the transmission of healthcare-associated respiratory infections [3,4,13]. Therefore, a cross-sectional survey was conducted to determine the prevalence (detectability) of the microbiological presence of the influenza virus among HCWs during the epidemiological season using novel POCT—the Flu SensDx device and to correlate it with the vaccination status and clinical symptoms of ILI.

## 2. Materials and Methods

### 2.1. Study Design

The cross-sectional survey was conducted between January and March 2020 in Wroclaw (the capital city of Lower Silesia region, Poland). The objective of this study was to estimate the value of the influenza virus infection ratio among participating HCWs by POCT performed by the Flu SensDx device. Molecular techniques were also used—part of the samples were additionally tested by RT-PCR as a control.

Nineteen public primary healthcare settings (PHCS) were pre-selected from all 153 PHCS in Wroclaw using systematic sampling and 3 hospitals were pre-selected from 6 main facilities using purposive sampling. All pre-selected PHCS and 2 of 3 hospitals accepted the invitation to participate in this study. Five or four wards per hospital were selected to participate, mainly internal-medicine and pediatric wards (patients at high risk of influenza). All personnel of the selected PHCS and hospital wards were set as the target population. Recruitment was performed by inviting all medical and non-medical staff to participate during personal visits of the principal investigator in selected healthcare facilities. Participation in the study was voluntary. Approval for distributing the invitation and questionnaire was obtained from the board of each healthcare facility participating in the survey. After receiving written information about the study and a brief oral description of the aim of the study, written informed consents were obtained from all of the participants before entering the study, and then the participants received self-administered standardized questionnaires to complete and throat and nasal swabs were collected.

**Figure 1:**
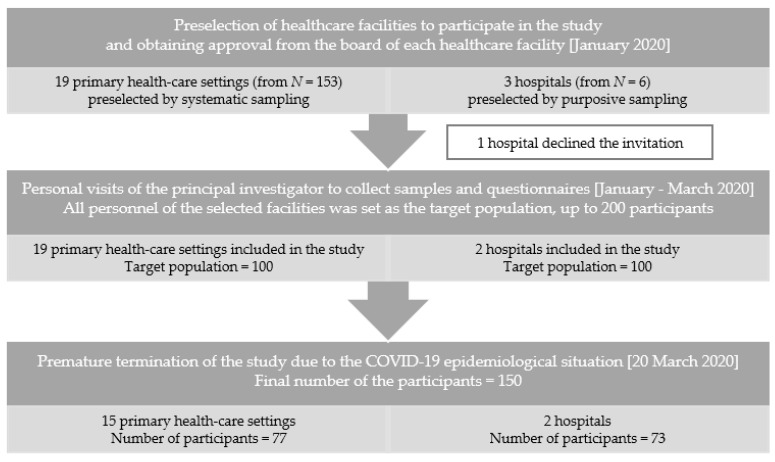
Flowchart of the recruitment procedure and study design.

The target number of participants was set at 200. The study was terminated prematurely due to the COVID-19 epidemiological situation. Eventually, HCWs from fifteen selected PHCS (10% of all PHCS in Wrocław) and part of selected hospital units (endocrinology, hematology, oncology, angiology and rheumatology units from the University Clinical Hospital and a pediatric intensive care unit from another multidisciplinary hospital) participated in the study with the final 150 number of participants. The recruitment procedure is presented in Figure 1.

### 2.2. Detection of Influenza Virus

Throat and nasal swabs were collected personally by the study principal investigator during visits to selected healthcare facilities. Swabs were collected in double—one pair was destined to perform a POCT, while the second pair was destined to perform a RT-PCR test. The study did not use nasopharyngeal swabs, which can be uncomfortable to collect for participants. Regularly flocked nylon swabs (Copan FLOQSwabs™, FLOQSwabs^®^, Copan Diagnostics, Inc., Murrieta, CA, USA) were used to collect all samples (for POC and RT-PCR testing).

POCT was performed by the Flu SensDx device (direct detection of M1 protein by EIS), directly after collection of each sample, according to the manufacturer’s instructions. The assay is performed by extracting the virus M1 protein from the swab with the use of a buffer and then sprinkling the extract on the microsensor. The MOBI SensDx (SensDx S.A., Gdansk, Poland) platform measures an interaction between the bioreceptor molecules and the M1 protein using EIS and initial results are transferred to the SensDx App (SensDx S.A., Gdansk, Poland) which, when installed on a Windows (Microsoft Corporation, Redmond, WA, USA) computer, presents the final result in the form of qualitative results: positive, negative or uncertain within up to 5 min.

Upon completing the collection of swabs after each visit, all samples were immediately transported in 2 mL of viral transport medium to a laboratory (maximum up to 4 h, up to 8 °C), where they were screened for influenza virus by RT-PCR testing. Total nucleic acid was extracted from samples by using the Total RNA Mini extraction system (A&A Biotechnology, Gdynia, Poland) according to the manufacturer’s instructions. To detect influenza virus, a reverse-transcription quantitative real-time PCR (RT-qPCR) assay was performed and SensiFAST™ SYBR^®^ No-ROX Mix (Bioline, London, UK) was used. The reaction of RT-qPCR was performed by CFX Connect Real-Time PCR Detection System (Bio-Rad Laboratories, Inc., Hercules, CA, USA). All reagents were certified for in vitro diagnostic use and were used according to the manufacturers’ guidelines. The forward primer (5′-AAGACCAATCCTGTCACCTCTGA-3′) and the reverse primer (3′-CAAAGCGTCTACGCTGCAGTCC-5′) were used for amplification of the matrix gene of influenza A virus and the forward primer (5′-GAGACACAATTGCCTACCTGCTT-3′) and the reverse primer (3′-TTCTTTCCCACCGAACCAAC-5′) were used for influenza B virus detection [14]. Cycling conditions were as follows: initial denaturation at 95 °C for 2 min, followed by 40 cycles of 95 °C for 5 s, 60 °C for 10 s, and 72 °C for 5 s. At the end of the assay, RT-qPCR products were subjected to a melting-curve analysis to determine the specificity of the assay. The products of the RT-qPCR reaction were described as positive (both criteria): (a) a melt temperature of approximately 82 °C for influenza A virus and 77.5 °C for the influenza B virus; (b) the quantification cycle (Cq) at <35. Products with Cq in the range of 35–38 were described as “uncertain” result, i.e., containing a trace of influenza virus genetic material. 

### 2.3. Study Questionnaire

The anonymous self-administered questionnaire was composed of two sections. The first section included socio-professional variables, such as demographic details (gender, age), occupational group, type of healthcare facility (primary healthcare settings/hospital) and years of experience. Occupational groups were categorized as physicians, nurses, allied medicals (for example, physiotherapists and laboratory diagnosticians) and non-medical staff (administration, cleaning and other support staff). Medical students were excluded from participating in this study. The second section assessed self-reported uptake of influenza vaccination in the 2018–2019 and 2019–2020 seasons and the presence of symptoms of ILI in the last 3 days (with multiple choice responses or free text field for other answers).

### 2.4. Outcome Measures

There was one main outcome measure in this study: the rate of indicating the influenza virus infection among participating HCWs determined by Flu SensDx testing. Additional research questions are: (1) is the influenza-positive result correlated with the declared influenza immunization status and (2) with the reported presence of symptoms of ILI among the survey group of HCWs?

### 2.5. Statistical Analysis

Upon the completion of data collection, the data were coded into categorical variables and double-checked. Descriptive statistics were generated for all survey items. The main analysis was the comparison of positive and negative respondents tested by the Flu SensDx device. Categorical variables were compared using Pearson’s *χ*^2^ tests of association with Yates’ continuity correction. The significance level was set at *p* = 0.05. The statistical analyses were performed using R version 3.6.3 statistical software (R Foundation for Statistical Computing, Vienna, Austria).

## 3. Results

### 3.1. Study Population

The study comprised 150 HCWs—a total of 150 pairs of samples (nasal and throat swabs, NTS) and completed questionnaires were collected. There were 83 (55.3%) physicians and 40 (26.7%) nurses in total. Females accounted for 82.7% of respondents, while approximately 27% of the respondents were aged over 50. Half of the participants worked in PHCS (51.3%) and years of job experience ranged from 1 to 43 years with a median of 15 years. Declared influenza vaccination coverage was 61.3% in 2019–2020 and 46.0% in the 2018–2019 season for all participants. There were no statistically significant differences between survey HCWs from PHCS and hospitals by their questionnaire-based socio-professional determinants and influenza vaccination 2018–2019 and 2019–2020 status (*p* > 0.05). The characteristics of the study participants are fully presented in Table 1.

### 3.2. Symptoms

More than half of the participants (54.7%; 82/150) did not report any symptoms, while the remaining 45.3% reported at least one symptom of ILI in the last 3 days before swab collection, including a cough (29.4%; 20/68) or fever (4.4%; 3/68). According to descriptive statistics, at least one symptom of ILI was more frequently reported by women, the youngest respondents (<50 years old), nurses (difference at 13.9 percent point compared to physicians), HCWs from PHCS or with shorter job experience, and participants who were vaccinated during the survey or the previous season (difference in range 0.8–1.9 percent point), however, there were no significant differences between (a)symptomatic HCWs and their socio-professional determinants or influenza vaccination 2018–2019 and 2019–2020 status (*p* > 0.05; data not shown). Almost half of asymptomatic HCWs tested positive by Flu SensDx (48.8%; 40/82), while 1/3 of symptomatic participants were tested positive (38.2%; 26/68). Among participants with fever, no one was tested positive and almost 1/3 HCWs who reported cough in the last 3 days were tested positive (6/20).

### 3.3. Detection of Influenza Virus

#### 3.3.1. Flu SensDx Testing

All the collected samples were tested by the Flu SensDx device. One throat sample was positive (<1%), while two were uncertain (1.3%) and the rest of the samples were negative (98.0%; 147/150). Almost half of the nasal samples were tested as positive (44.0%; 66/150), while the rest of them were negative and “uncertain” results were not obtained. Therefore, only results performed on nasal samples were analyzed. According to descriptive statistics, a positive result was obtained more frequently in: women, the youngest respondents (<50 years old), nurses, HCWs from PHCS, or with shorter job experience, however, all the mentioned variables were statistically independent (*p* > 0.05)—Table 1. Among positively tested participants, 60.6% (40/66) were asymptomatic, and 63.6% (42/66) were influenza vaccinated in the survey season, but each variable was not statistically significant (*p* > 0.05). Approximately 1/3 of positively tested participants (36.6%; 24/66) were both asymptomatic and vaccinated. Among symptomatic and positively tested participants, 23.1% of them reported cough (6/26) and nobody reported fever (0/26).

#### 3.3.2. PCR Testing

16.7% of NTS (25/150) and an additional 18.4% of the nasal samples (23/125) were screened by molecular technique (RT-qPCR), and a total of 32% (48/150) of nasal samples were screened (linked to 55% of positive and 14% of negative Flu SensDx samples). Two throat (linked to positive and uncertain results of Flu SensDx testing) and 60.4% (29/48) of nasal samples were positive. Full coherence between POC and RT-PCR results (fraction of true positive and negative results) was achieved in 96.0% of tested by the throat (1 case of mismatch was related to a positive RT-PCR result linked to an uncertain Flu SensDx result) and 81.3% of nasal samples. Including “uncertain” RT-PCR results (i.e., detection of a trace of influenza virus material) as a “weak” positive, the coherence level rose to 87.5% (nasal samples)—Table 2. At the same time, 78.4% (29/37) of positively tested samples by Flu SensDx were confirmed by RT-PCR testing (positive predictive value, PPV) and using the model which included a “weak” positive RT-PCR results PPV was at 97.3% (36/37). Influenza A virus was detected in all positively tested samples (*N* = 31), including 2 nasal samples which were also positive for influenza B virus. A trace amount of influenza B virus was additionally detected in 1 throat and 12 nasal positives for influenza A samples. Including nasal samples with the detected trace amount of viral material only (extended model of RT-PCR results), 8 of them were positive for influenza A virus, while 3 were positive for influenza B virus. Due to a small number of the performed RT-PCR assays, the sensitivity and specificity of Flu SensDx testing related to RT-PCR results were not calculated. Among RT-PCR positively tested HCWs (*N* = 29; mentioned 2 positive throat samples were linked to participants with RT-PCR positive nasal samples also), 69.0% were influenza vaccinated in the survey season and 55.2% were asymptomatic, but each variable was not statistically significant (*p* > 0.05).

## 4. Discussion

The current study is preliminary and exploratory. In our opinion, the main results of the present study are very interesting and worthy of further wide discussion. Almost half of the participated HCWs were positively tested for influenza by quick influenza test (the Flu SensDx device; nasal swabs as diagnostic material) with moderate-to-high coherence with results of RT-PCR testing. Although positive results of the testing were obtained more frequently among HCWs (paradoxically) with a positive status of survey season influenza vaccination or with the absence of ILI symptoms at least 3 days before swab collection, it was not a statistically significant correlation in terms of each variable. It is worth highlighting that only one in 150 throat samples tested by Flu SensDx was positive. In our opinion, there are two possible explanations for this result. The first case—swabs were collected correctly and were truly free of influenza virus material or the second case, less probable in our opinion, swabs were incorrectly collected and therefore were false negative. All of the samples were collected by the principal study investigator, who is a physician by profession, so we assume that the swab collection technique was fully correct. It is worth highlighting that RT-PCR testing confirmed the mentioned case as a positive result. What’s more, the result of RT-PCR testing of throat samples was a “weak positive” (a trace amount of viral material) in 6 cases (Table 2), which may additionally confirm the fact that the material was collected correctly. Despite this, we could not fully exclude the second option because our RT-PCR testing protocol was not included an internal control, i.e., detection of so-called housekeeping genes (e.g., beta-actin mRNA) [15,16]. Therefore, the results of the nasal swabs only were analyzed as more reliable.

### 4.1. Asymptomatic Presentation of Influenza Infection

Efficient strategies of limiting the spread of influenza require reliable estimates of the rate of people with an asymptomatic presentation of infection and their contribution to the virus transmission chain, both in communities and healthcare facilities [17,18], but people with asymptomatic or mild influenza illness have rarely been systematically investigated [19]. Therefore, systematic laboratory epidemiological surveillance, independently of the definition of a clinical (symptomatic) case, would be more appropriate because it would also allow the recording of asymptomatic cases [20]. The current policies arbitrarily assume a constant rate of asymptomatic infection in the range of 30–50% [17]. The authors of a systematic review and meta-analysis of 55 studies (PubMed and Web of Science database; up to 2015) reported that overall, the prevalence of laboratory-confirmed asymptomatic influenza infection ranged from 5.2% to 35.5% with a pooled rate of 19.1% for any type of influenza [17]. Similarly, the authors of another systematic review and meta-analysis of 30 studies (PubMed and Scopus database; up to April 2014) estimated the fraction of asymptomatic laboratory-confirmed cases at 4–28% with a pooled mean of 16% [21]. It is highly possible that substantial cases of influenza virus infections are underestimated in general, mainly in terms of asymptomatic presentation and it may depend on the method which was used to identify the infection (sera/swabs testing). The same authors reported, based on longitudinal studies performed by testing of sera, that the asymptomatic fraction adjusted for illness from other causes fell in the range 65–85% and it was higher than most of the unadjusted estimates. In our opinion, Ip et al., reported a very simple and obvious, but important and memorable observation, i.e., some of the symptoms of ILI, like runny nose or cough, could have a cause other than influenza and other than ARI, e.g., poor air quality or allergies and therefore it could lead to underestimating the fraction of asymptomatic infections [19]. More explanations were obtained in a study whose authors used a novel log-linear binomial regression model and serological data from Taiwan (the 2005–2006 influenza epidemic season; 1007 children) [22]. The results showed that the adjusted pathogen-specific asymptomatic ratios based on the mathematical model were higher than raw data based, i.e., 0.75 and 0.65 for H1N1 and H3N2 influenza virus infection vs. 0.65 and 0.57, respectively. What’s more, the authors using data from another study (Boivin et al., 2000) with the raw asymptomatic ratio obtained at 22%, reported that the model-based asymptomatic ratio for influenza could be up to 40%. A higher ratio of asymptomatic influenza infection among the participating HCWs in the current study in comparison to results of the above community studies could be explained by results of a systematic review and meta-analysis of the incidence of influenza among HCWs and other healthy adults by Kuster et al., (58,245 participants in total; influenza seasons 1957–2009), which showed that medical personnel faces a higher risk of influenza infections compared to the population of healthy adults working in non-healthcare facilities [4]. The authors also attempt to explain the higher rates of asymptomatic influenza infection among medical personnel in comparison with other healthy non-HCWs and hypothesize that HCWs, who are more exposed to influenza infections (including post-vaccination exposure), develop more effective immunity mechanisms that reduce the severity of infection symptoms.

### 4.2. Symptomatic Presentation of Influenza Infection

It is not known if the level of viral shedding perfectly correlates with the risk of influenza transmission. The empirical data showed that the intensity of influenza ribonucleic acid (RNA) shedding was rather correlated with the intensity of symptoms, especially fever [11,15,19,23,24], but the viral shedding was detectable also in the asymptomatic and paucisymptomatic cases [8,19,24,25]. It suggests the potential for influenza virus transmission even in the absence of clinical symptoms, however, it is possible that certain symptoms play a key role in infectivity (transmission). Although various authors in the literature point out that the detection of viral RNA could be a reasonable indication of viral shedding and infectiousness (for example, Ip et al., showed a strong correlation between shedding loads of influenza viruses [RT-PCR testing] and virus infectivity measured by quantitative viral culture assays [24]), but detection of viral RNA is not the same as isolation of infectious viruses (PCR cannot differentiate between non-infective viral nucleic acid and infective virion) [19]. It is worth highlighting that determining infection based on PCR testing only may lead to underdiagnosing of some cases, e.g., Leung et al. reported that some persons could have serologic evidence of influenza infection with PCR-based negative test (some infected patients not shedding virus or shedding at such low loads that they were not detectable by molecular testing [PCR]) or without any symptoms and vice versa [21]. However, it cannot be denied that influenza positive-tested employees without fever may shed virus and therefore pose a risk of influenza transmission to patients or coworkers [26]. In the present study, nobody positive-tested HCWs (based on nasal samples) reported fever and almost 10% of them reported a cough at least 3 days before swab collection. However, in the present study, two independent methods to detect the influenza virus material (M1 protein and viral genetic material) were used and due to the obtained moderate-to-high level of coherence between positive results of Flu SensDx and RT-PCR testing (up to 97.3%, depending on the used model of RT-PCR results—Table 2), we can assume the presence of completed virions of influenza in the analyzed material. However, the infectiousness of these samples was not verified (e.g., by viral culture).

### 4.3. Strategy to Influenza Infection Control

Although the role of the asymptomatic or afebrile people (HCWs) in influenza virus transmission is uncertain, the main question is what type of strategy should be implemented to reduce the risk of nosocomial influenza infections. On the one hand, the empirical data have shown clearly a correlation between viral RNA shedding loads and the presence of symptoms (influenza A virus infections), which may assume the implementation of a symptoms-based strategy [24]. Therefore, non-pharmacological interventions, such as wearing protective masks or hand hygiene, should be effective in preventing the transmission of infection in community or healthcare settings. Interestingly, the authors of the Cochrane group’s updated review of physical interventions to interrupt or reduce the spread of respiratory viruses (67 studies in total, up to April 2020; no studies conducted during the COVID-19 pandemic) reported that there is (1) low and (2) moderate certainty evidence that wearing a mask (1) may make little or no difference to the outcome of ILI and (2) probably makes little or no difference to the outcome of laboratory-confirmed influenza, compared to not wearing a mask (two trials with HCWs and seven in the community) [27]. In conclusion, the pooled results of randomized trials did not show a clear reduction in respiratory viral infection with the use of medical/surgical masks during seasonal influenza. However, data showed that hand hygiene may offer a benefit with the 11% relative reduction of respiratory illness (low-certainty evidence). Based on the early experiences of the COVID-19 pandemic in Poland (March–May 2020), it could be assumed that physical distancing is a very efficient intervention—according to the data of the National Institute of Public Health-National Institute of Hygiene (pol. Narodowy Instytut Zdrowia Publicznego-Państwowy Zakład Higieny, NIZP-PZH) there was a drastic decrease in the incidence of influenza and ILI in official statistics, as a consequence of the introduction of a hard lockdown [28]. According to the authors’ own analysis (based on NIZP-PZH data), 34.8% less cases were noted of the total number of influenza and ILI in the period from 1 March to 31 May in 2020 compared to 2019 (764,293 vs. 1,171,718 cases), including a spectacular reduction at a level of 69.9% cases in the period from 1 April to 31 May (182,491 vs. 605,656). However, physical distancing could be successfully implemented in the community as a prevention method, but it could be difficult or impossible to implement in healthcare facilities, where direct contact with a patient is often necessary. On the other hand, describing the bimodal course with an early peak and a prolonged period of viral shedding in cases of influenza B virus infections may imply the potential infectiousness before the onset of symptoms and after clinical improvement [24]. It is worth highlighting that the influenza B virus caused a substantial proportion of influenza infections globally in the 21st century [29] and for example, the type B virus of the Yamagata lineage predominated in the 2017–2018 season in Poland [30]. What’s more, in the current study, including a trace amount of detected viral material, the influenza B virus was detected in almost half of the positively tested nasal samples. Additionally, the laboratory data showed that among patients with seasonal A(H3N2) influenza, mean viral RNA loads were at comparable levels regardless of the presence or absence of symptoms [19]. These facts show the possibility of influenza virus transmission from infected people (patients/HCWs) despite the absence of clinical symptoms and imply the hypothesis that people can be a source of influenza infection before they become clinically ill or after clinical improvement [24]. Having this in mind, the mentioned non-pharmacological interventions could be inadequate, and generally preventive measures, including good general hygiene practices, would not be as effective as influenza vaccinations to control the influenza virus infection [19]. Unfortunately, the vaccination rates in the general population vary in different areas of the world, and for Poland they are extremely low, at around 3.5% (data from 2008–2018 [30]), and similarly, the influenza vaccination rates among HCWs are universally low (2–44% globally) and vary over time as well as between regions and different types of healthcare professionals (physicians/nurses) [31]. In the present study, the influenza vaccination coverage was 61.3% in 2019–2020 and 46.0% in the 2018–2019 season for all participants. There is an urgent need to implement well-organized campaigns to increase vaccination rates among HCWs as well as in the case of the general population—issues of HCWs influenza vaccination (vaccination coverage, determinants, possible interventions and importance of influenza vaccination) were widely discussed in other authors’ publications.

### 4.4. Influenza Vaccination and Influenza Infection

Despite the obvious benefits of influenza vaccination in reducing the incidence of infection, there are debatable data as to whether HCWs’ influenza vaccination reduces the incidence of influenza infections among patients [3,32,33]. It’s worth highlighting the fact that among positive tested participants by the Flu SensDx device, most of them were influenza vaccinated in the survey season, but it was not a statistically significant correlation (*p* > 0.05). It is worth quoting here an interesting analysis of the testing of hospital HCWs with respiratory symptoms (January–February 2014, *N* = 449), with a result of 54% of employees who had a positive test for any respiratory pathogen, including 9.1% HCWs tested positive for influenza [26]. Among all influenza-infected HCWs, 51.2% had a fever (21/41) and 52.6% had previously received influenza vaccination (20/38; 3 participants had an unknown vaccination status). Interestingly, there was no significant difference in influenza-infected HCWs with febrile and their influenza vaccination status. Similarly, in a German study (epidemic season 2014–2015, 677 participants), 24% of hospital staff reported the occurrence of ARI during the infection period (83% reported coughing), of which 9% of staff reported an ILI, defined as fever ≥38.5 °C and the sudden appearance of symptoms [13]. The study did not demonstrate a statistical relationship between the reported probable influenza infection and immunization status (authors reported possible selection bias). It is worth highlighting that the influenza virus triggers a very complex immune response and it is still unclear whether influenza vaccination may be able to fully block the chain of transmission, or whether it simply reduces the severity of the disease in vaccinated subjects. A commonly used inactivated influenza vaccine (IIV; 89.6% of global production of seasonal influenza vaccines in 2019) leads to the production of neutralizing serum antibodies in contrast to a live attenuated influenza vaccine (LAIV, mucosal administration; 5.0% of global production respectively) which leads to the production of both serum and mucosal antibodies [34,35]. According to the results of animal studies, efficacy in blocking the horizontal transmission of the influenza virus was much better for LAIV (as a possible effect of mucosal antibodies), however, IIV led to the reduction of the viral load after the challenge and partially reduced the number of secondary transmission cases [36]. It is worth pointing out that LAIV is available in Poland but registered for use for patients < 18 years old only, therefore only IIV can be used for vaccination of HCWs. The mechanism of the IIV may explain our result of a relatively high ratio of influenza positive-tested HCWs, including an asymptomatic fraction, regardless of the immunization status, and its statistical independence (*p* > 0.05).

### 4.5. Practical Implications

In our opinion, keeping in mind all of the above considerations and results of this study, it would be reasonable to consider the wide implementation among HCWs of both mentioned non- and pharmacological interventions to reduce the risk of influenza (and other respiratory viruses) nosocomial transmission. Due to the current COVID-19 pandemic, commonly wearing face masks and hand hygiene are already implemented, but we are not sure about the implementation of vaccination among HCWs, especially against influenza. Although the benefits of HCW influenza vaccination for patients are still inconsistent and widely discussed [3], in the authors’ opinion and given the safety, effectiveness and other possible benefits of influenza vaccines, all efforts to increase influenza vaccination rates among HCWs are reasonable, especially in the era of the COVID-19 pandemic. The above recommendations take on even more meaning due to the fact that HCWs are a professional group with a significant level of presenteeism, up to 75% [7]. In the present study, the presenteeism rate was 45.3% for all medical workers, including 38.6% for physicians and 52.5% for nurses (!) (data not shown).

These results deserve further attention with regard to ensuring universal infection prevention precautions, irrespective of symptomatic and immunization status, and clearly confirm that our knowledge is uncertain and incomplete. Referring to considerations by Tiwari et al. [37], we sum up as below:

- HCWs are at high risk of exposure to influenza, SARS-CoV-2 and other respiratory viruses. Undiagnosed HCWs can transmit the above viruses to patients and pose an occupational hazard to coworkers.
- There are reported cases of prolonged influenza virus shedding among asymptomatic individuals. It is unclear what is the contribution of positive tested, but asymptomatic people (HCWs) to the transmission chain. There are challenging questions, for example when the asymptomatic but influenza positive-tested HCWs should be allowed to return to work. Finding an answer to this question is important to ensure a safe environment for patients and other HCWs, especially when faced with staff shortages.

### 4.6. Strength and Limitations

To the best of our knowledge, this is the first study aimed to assess the rate of laboratory-confirmed influenza infection among Wroclaw HCWs. In addition, two independent methods of detecting influenza virus material (M1 protein and viral RNA) and the innovative Flu SensDx device were used to supplement general data.

There are several limitations to this study. Firstly, selection bias is possible and due to this fact, the study sample may not be fully representative for HCWs in Wroclaw city. Our ability to generalize the findings from this study is limited because we cannot compare the survey population with the general one due to the lack of a full list of HCWs from all Wroclaw PHCS and hospitals. In addition, this survey was conducted mainly among HCWs from one hospital and only 10% of PHCS in Wroclaw during one influenza season —it reflects the part of the current influenza infection status and does not describe changes across time. In fact, our sample size of 150 may not be large enough to be statistically significant to identify small-to-moderate associations. Secondly, it is advisable to conduct a similarly designed study in the future with a larger number of participants, using a more representative sample of HCWs and to extend a testing protocol to verify all (or most) of the collected Flu SensDx samples by RT-PCR testing. It is worth including an internal control with detection of housekeeping genes in the RT-PCR testing protocol and using viral culture as an additional method to assess infectivity of collected samples and correlate it with POC/RT-PCR testing results and other variables like an influenza vaccination status and the presence of symptoms.

## 5. Conclusions

Larger studies are needed to estimate the incidence of influenza virus infection among HCWs, especially with regard to the implementation of physical barriers and to ascertain the duration of viral RNA persistence among (a)symptomatic HCWs to understand the underlying mechanism and fine-tune some policies aimed at the reduction of nosocomial infections, including influenza virus infection.

## Figures and Tables

**Table 1:** Characteristics of the study participants.

Characteristics	Total ^a^ (*N* = 150)	PHCS ^a^	Hospital ^a^	*p*-Value *	Positive Result by Flu SensDx ^a^ (*N* = 66; 44.0%)	IIR	*p*-Value *
Gender							
Female	124 (82.7)	66 (86)	58 (79)	0.425	55 (83.3)	44.4	1
Male	26 (17.3)	11 (14)	15 (21)		11 (16.7)	42.3	
Age Group (Years)							
≤50	110 (73.3)	51 (66)	59 (81)	0.067	49 (74.2)	44.5	0.970
>50	40 (26.7)	26 (34)	14 (19)		17 (25.8)	42.5	
Occupational							
Physicians	83 (55.3)	38 (49)	45 (62)	0.122 ^d^	38 (57.6)	45.8	1 ^d^
Nurses	40 (26.7)	25 (33)	15 (20)		19 (28.8)	47.5	
Allied Medical Staff ^b^	14 (9.3)	9 (12)	5 (7)		6 (9.1)	42.9	
Nonmedical Staff ^c^	13 (8.7)	5 (6)	8 (11)		3 (4.5)	23.1	
Job Experience (Years)							
≤10	59 (39.3)	24 (31)	35 (48)	0.053	26 (39.4)	44.1	1
>11	91 (60.7)	53 (69)	38 (52)		40 (60.6)	44.0	
2019–2020 Influenza Immunization							
Yes	92 (61.3)	48 (62)	44 (60)	0.927	42 (63.6)	45.7	0.730
No	58 (38.7)	29 (38)	29 (40)		24 (36.4)	41.4	
2018–2019 Influenza Immunization							
Yes	69 (46.0)	41 (53)	28 (38)	0.096	33 (50.0)	47.8	0.480
No	81 (54.0)	36 (47)	45 (62)		33 (50.0)	40.7	
Location of Work							
Primary Health-Care Setting	77 (51.3)	-	-	-	37 (56.1)	48.1	0.389
Hospital	73 (48.7)	-	-	-	29 (43.9)	39.7	
Symptoms							
0	82 (54.7)	39 (51)	43 (59)	0.395	40 (60.6)	48.8	0.258
≥1	68 (45.3)	38 (49)	30 (41)		26 (39.4)	38.2	

PHCS—primary health-care settings, IIR—influenza infection rate (%). ^a^ Values are presented as *N* (%); ^b^ Physiotherapists, laboratory diagnosticians; ^c^ Administrative, cleaning and supporting staff; ^d^ The Pearson’s χ^2^ test was calculated only for a group of physicians and nurses due to the small number of other categories; * for Pearson’s Chi-squared test with Yates’ continuity correction.

**Table 2:** Statement of results performed by Flu SensDx and RT-PCR.

	Standard Model of RT-PCR Results	Extended Model of RT-PCR Results (A Trace Viral Genetic Material)
Number of Samples Tested by RT-PCR	The Fraction of True Positive and Negative Results	
Throat Samples (*N* = 25)	96.0% (24)	72.0% (18)
Nasal Samples (*N* = 48)	81.3% (39)	87.5% (42)
Total (*N* = 73)	86.3% (63)	82.2% (60)
Number of Samples Positively Tested by the Flu SensDx Device	Positive Prediction Value of the Flu SensDx Device	
Throat Samples (*N* = 1)	100.0% (1)	100.0% (1)
Nasal Samples (*N* = 36)	77.8% (28)	97.2% (35)
Total (*N* = 37)	78.4% (29)	97.3% (36)

NOTE: Values are presented as % (*N*); RT-PCR, real-time reverse transcription polymerase chain reaction.

## Data Availability

The study is registered on ClinicalTrials.gov (NCT04223544).
